# Functional Magnetic Resonance Imaging in Conscious Animals: A New Tool in Behavioural Neuroscience Research

**DOI:** 10.1111/j.1365-2826.2006.01424.x

**Published:** 2006-05

**Authors:** CF Ferris, M Febo, F Luo, K Schmidt, M Brevard, JA Harder, P Kulkarni, T Messenger, JA King

**Affiliations:** Center for Comparative Neuroimaging, Department of Psychiatry, University of Massachusetts Medical School Worcester, MA, USA

**Keywords:** fMRI, BOLD, cerebral blood volume, cerebral blood flow, functional imaging, neuroanatomy, magnetic resonance

## Introduction

Functional magnetic resonance imaging (fMRI) is a unique window to the brain, enabling scientists to follow changes in brain activity in response to hormones, ageing, environment, drugs of abuse and other stimuli. There are two features that make fMRI unique when compared with other imaging modalities used in behavioural neuroscience. First, it can be entirely noninvasive: each animal can serve as its own control over the natural course of its life, vital for following neuroadaptation and other developmental processes critical to understanding behaviour. Second, fMRI has the spatial and temporal resolution to observe patterns of neuronal activity across the entire brain in less than a minute. Although fMRI does not have the cellular spatial resolution of immunostaining, nor the millisecond temporal resolution of electrophysiology, synchronised changes in neuronal activity across multiple brain areas seen with functional MRI can be viewed as functional neuroanatomical circuits coordinating the thoughts, memories and emotions for particular behaviours. Thus, fMRI affords a systems approach to the study of the brain, complementing and building from other neurobiological techniques to understand how behaviour is organised across multiple brain regions. In this review, we present a general background to fMRI and the different imaging modalities that can be used in fMRI studies. Included are examples of the application of fMRI in behavioural neuroscience research, along with discussion of the advantages and disadvantages of this technology.

## What are the different fMRI techniques and how do they work?

### Blood oxygen level dependent (BOLD) technique

**FIG. 1:**
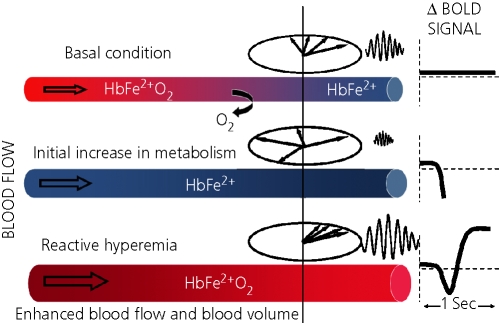
Schematic diagram showing conditions contributing to blood oxygen level dependent (BOLD) signal changes (31).

Functional MRI indirectly detects neural activity in different parts of the brain by comparing contrast in MR signal intensity prior to and following stimulation. What is responsible for the change in MR signal intensity? Areas of the brain with increased synaptic and neuronal activity require increased levels of oxygen to sustain the heightened metabolic activity involved. Enhanced brain activity is accompanied by an increase in metabolism followed by local increases in blood flow and blood volume (1–4). The enhanced blood flow usually exceeds the metabolic demand, which exposes the active brain area to a high level of oxygenated haemoglobin. This is important for BOLD imaging because oxygenated haemoglobin increases the MR signal intensity, whereas deoxygenated haemoglobin decreases MR signal intensity. The relationship between metabolism, haemoglobin oxygen saturation, blood flow and MR signal intensity is shown in Fig. 1 and is called the blood oxygen level dependent technique for functional imaging or BOLD (5).

BOLD depends on the different MR signal intensities associated with oxygenated versus deoxygenated haemoglobin. So how does haemoglobin oxygenation affect he MR signal? To understand this we first need to deal with the basis of the MR signal. Mobile protons associated with hydrogen atoms in water and fat are the primary source of MR signal. The hydrogen nucleus with its single charged proton spins creating a surrounding electromagnetic field with the characteristics of a magnetic dipole. When placed in an external magnetic field, quantum mechanics tells us that this hydrogen nucleus can have two ‘spin’ states or energy levels. Most of the nuclei prefer the lower energy state rather than the higher. When electromagnetic radiation in the radiofrequency range (MHz) is applied, nuclei can absorb energy and be elevated to the higher energy state. The energy given off as these nuclei ‘relax’ back into their lower state is the source of the MR signal.

**FIG. 2:**
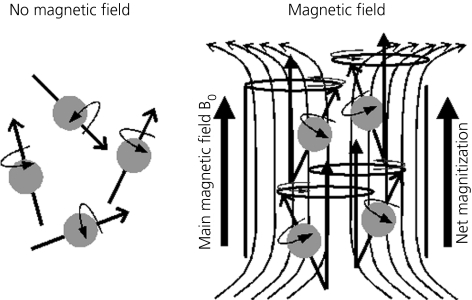
Schematic diagram showing the behaviour of hydrogen nuclei in magnetic and nonmagnetic environments (31).

A more intuitive but less accurate description of nuclear magnetic resonance is provided by classical mechanics. Consider each hydrogen nucleus as a spinning top with a random orientation in a non magnetic environment as shown in Fig. 2. When placed into a magnetic field, the hydrogen nuclei align in parallel with the field lines of the magnet. The net magnetisation from all of these aligned nuclei is shown as a vector parallel to the main external magnetic field B_0_. These aligned nuclei are the source of potential energy for MR signal. Increasing the strength of the magnetic field recruits more hydrogen protons for imaging. The differences in hydrogen proton density between tissues like cerebrospinal fluid, grey and white matter are one reason for signal contrast in neuroanatomical imaging.

**FIG. 3:**
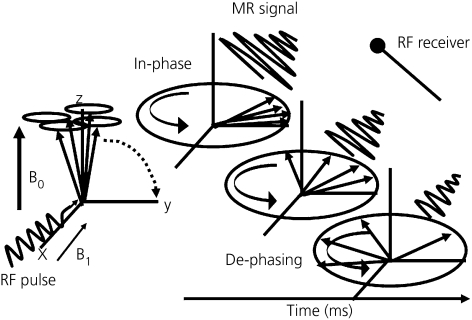
Schematic diagram showing behaviour of hydrogen nuclei flipped into the transverse x–y plane with a radiofrequency (RF) pulse. B_0_ is the main magnetic field and B_1_ is the magnetic field created in the transverse plane by the RF pulse (31).

The spinning hydrogen nuclei ‘wobble’ or show an angular precessional frequency when placed in the main magnetic field as depicted by the circles in Figs 2 and 3. This precessional frequency is directly related to the field strength of the magnet. Animal magnets with field strengths of 4.7, 7.0, 9.4 and 11.7 Tesla create precessional frequencies for hydrogen nuclei of 200, 300, 400 and 500 MHz, respectively. Brief radiofrequency (RF) pulses can be used to create a magnetic field B_1_ perpendicular to the main field B_0_ as shown in Fig. 3. If these RF pulses have the same frequency as the precessing hydrogen nuclei then the resonant energy is absorbed, flipping the nuclei into the transverse x-y plane where they continue to precess. When initially flipped, all of the hydrogen nuclei precess at the same frequency and are said to be in-phase. This ‘in-phase’ precession in the transverse plane sets up an oscillating MR signal that can be picked up by a special antenna called an RF receiver or probe. However, this oscillating signal rapidly decays lasting only 20–50 ms. Interactions between these nuclei and inhomogeneties in their surrounding microenvironment cause some to precess slower than others resulting in ‘de-phasing. The BOLD technique enhances the MR signal because deoxygenated haemoglobin is paramagnetic and creates its own micromagnetic field, promoting de-phasing and thus reducing MR signal (Fig. 1). However, oxygenated haemoglobin has very little magnetic susceptibility. Because active brain areas have enhanced blood flow and are high in oxygenated haemoglobin, phase coherence is promoted resulting in a stronger MR signal.

Following an RF pulse, hydrogen nuclei undergo two simultaneous events. They precess in the transverse plane generating MR signal for as long as they remain in-phase and they relax back into the orientation of the main magnetic field returning to their lowest energy state. Both time courses are exponential functions. The time constant for relaxation is called T_1_ whereas the time constant for de-phasing is called T_2_. T_1_ is measured in seconds and T_2_ is measured in milliseconds. A third time constant called T_2_* is shorter than T_2_ and reflects de-phasing caused by conditions in the microenvironment contributing to magnetic susceptibility. Changes in T_2_* are the bases behind BOLD signal changes in functional imaging.

It is important for MR imaging to note that, for as long as there is net magnetisation in the transverse plane, there is potential for generating a series of MR signals. Spin echo and gradient echo pulse sequences have been developed to bring the hydrogen nuclei back into phase or have them ‘echo’ repeatedly until the system comes back to equilibrium.

**FIG. 4:**
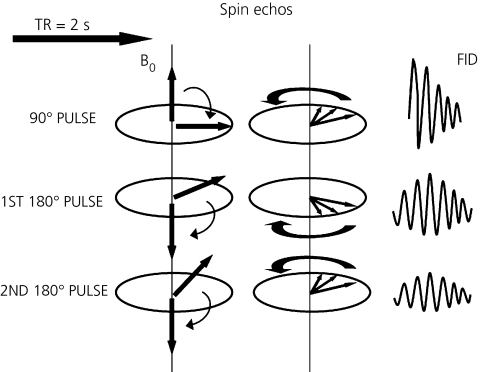
Schematic diagram showing the behaviour of precessing hydrogen nuclei in a spin echo pulse sequence. Net magnetisation (red arrows) in the main magnetic field (B_0_) slowly relaxes back to equilibrium over successive 180° pulses following the initial 90° pulse. Time to repeat (TR) is the repetition time between 90° pulses (31).

In a spin echo pulse sequence, the initial 90° pulse flips the hydrogen nuclei into the transverse plane as shown in Fig. 4. Afterward, a refocusing 180° pulse is applied, which reverses the direction of the precessional spins. The faster spinning hydrogen nuclei that have been turned around by these pulses are now facing the slower spinning nuclei. The MR signal waxes and wanes as the faster nuclei catch up and pass the slower nuclei. The 180° refocusing pulse can be applied several times while there is transverse magnetisation. Usually, after 1.5–2.5 s, the sequence is repeated (time to repeat or T_R_) and another 90° pulse is applied to flip the hydrogen nuclei into the transverse plane and the echo series begins again.

**FIG. 5:**
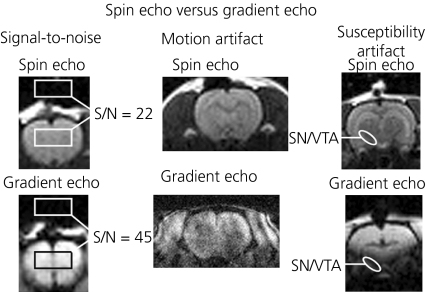
Shown are magnetic resonance images highlighting the advantages and disadvantages of spin echo and gradient echo pulse sequences. All images were collected from the same animal over the same imaging session. Susceptibility artifact is very pronounced in the substantia nigra (SN); and ventral tegmental area (VTA). S/N, Signal-to-Noise ratio (28).

One of the major advantages of fast spin echo pulse technique, particularly in imaging fully conscious animals, is its tolerance to artifacts from physiologic motion (e.g. cerebrospinal fluid movement) or magnetic susceptibility (e.g. distorted signal at air/liquid interfaces) (Fig. 5). However, magnetic susceptibility caused by deoxygenated haemoglobin is a key component in the BOLD signal. Hence, the fast spin echo technique is less sensitive to changes in BOLD signal than other techniques (see description of gradient echo, below). This problem of sensitivity can be addressed with higher field strengths and better RF electronics as discussed later.

**FIG. 6:**
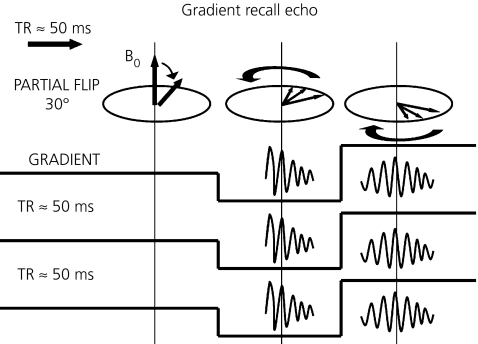
Schematic diagram showing the behaviour of precessing hydrogen nuclei partially flipped into the transverse plane at an angle of 30°. During the short-lived event, the gradient coils are used to reverse the magnetic field (black horizontal line) to refocus the precessing nuclei (31). TR, Time to repeat.

In a gradient echo pulse sequence, the initial RF pulse only partially flips the hydrogen nuclei into the transverse plane as shown in Fig. 6. Because these nuclei are only partially flipped, they rapidly lose their transverse magnetisation as they return to equilibrium in the main magnetic field. Hence, a series of short T_R_s usually between 50 and 75 ms are necessary to collect multiple MR signals. The main purpose of the gradient echo technique is to increase the speed of the scan. The rephasing event required for echo generation is accomplished by reversing the local magnetic field with special gradient coils. Gradient echo is particularly sensitive to magnetic susceptibility and, for this reason, is commonly used in functional imaging, where the differential magnetic susceptibility of oxygenated versus deoxygenated haemoglobin provides the signal. However, this advantage of increased sensitivity is also the major disadvantage of gradient echo imaging because of susceptibility artifacts, most noticeably seen in neuroimaging at the air/tissue interfaces associated with sinuses. Gradient echo is also very sensitive to shimming (improving the field homogeneity), making it difficult to collect undistorted slices across the entire brain.

### Cerebral blood flow (CBF) technique

Changes in image intensity due to the BOLD effect are the result of complex interactions between blood flow, blood volume, haemoglobin oxygenation and neurovascular coupling in metabolically active areas. However, a robust increase in BOLD signal can be obtained without a change in neuronal activity. A good example of this ‘uncoupling’ is a simple carbon dioxide (CO_2_) challenge. Inhalation of CO_2_ causes direct cerebrovascular dilation, increasing blood flow and BOLD signal intensity to brain tissue independent of metabolism (6). Indeed, theoretically, there is no change in cerebral metabolism with CO_2_ challenge. Consequently, it is not always true that a localised change in BOLD image intensity is a reflection of a change in brain activity. In these situations, MRI techniques which permit the noninvasive measurement of absolute or relative changes in CBF enable direct observation of haemodynamic changes without the confounding influence of deoxygenated haemoglobin.

**FIG. 7:**
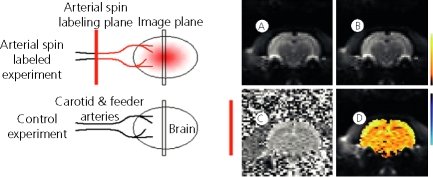
Schematic diagram showing arterial spin labelling procedure and image processing. (a) Control image. (b) Image acquired with arterial spin labelling. (c) Label/Control − 1. (d) False coloured overlay of blood flow. Note the enhanced blood flow to the cortex as compared to subcortical areas. Legend scale 0 ± 12%.

Noninvasive MRI techniques for measuring CBF use the protons in water molecules in the blood as an endogenous tracer, whereby the state of blood magnetisation is modified to produce a measurable change in signal intensity downstream in the tissue into which this blood flows (7). Two types of these arterial spin labelling techniques are commonly used in research, continuous arterial spin labelling (CASL) and pulsed arterial spin labelling (8). The CASL technique (Fig. 7) is commonly used in animal research applications, and requires two brain scans to be performed. In the first scan, an RF pulse is applied for a period of several seconds in a slice containing the carotid arteries. Blood flowing through these arteries at this location experiences complete inversion, and flows into the brain where these labelled water molecules in the blood exchange with water molecules in the brain. An image of the brain is then acquired in the presence of these labelled water molecules. In the second scan, the same arterial spin labelling pulse is applied to a location symmetrically opposed to the imaging slice, away from the feeding arteries. The same image is acquired in the absence of any labelled water molecules from arriving blood, and the difference between the control and labelled images yield a measure of local perfusion.

The CBF technique is widely used in imaging studies such as ischemic stroke to assess blood flow to the different areas of the brain; however, it has not displaced other imaging techniques widely used by behavioural neuroscientists to assess functional changes in brain activity. Because of the small amount of contrast (typically only a few percent of total image intensity between the control and labelled images), the sensitivity of arterial spin labelling CBF measurements is inherently lower than that of several other functional MRI modalities (9). Additionally, multislice CBF measurements using the CASL technique require that an additional coil, located near the carotid arteries supplying the brain, be used to transmit the labelling pulse (10). The limited sensitivity and complex hardware requirements of ASL experiments explain the comparatively infrequent use of this technique in the literature. However, CBF measurements can provide valuable information under certain circumstances. For example, when BOLD and CBF changes are measured simultaneously during the presentation of a stimulus, a calibrated calculation of changes in Cerebral Metabolic Rate of Oxygen consumption (CMRO_2_) can be performed, providing potentially enhanced inference into changes in the underlying neural metabolism than is possible using either measurement alone (11). In our own laboratory we are using CBF to assess baseline conditions prior to and following an imaging experiment because studies have shown that changes in CBF will alter the magnitude of the BOLD response (12–14). Evoked cortical responses produce greater changes in BOLD signal with low CBF and lesser changes with higher CBF. By assessing CBF over the course of a BOLD imaging study, we can better evaluate the quality of the data.

### Cerebral blood volume (CBV) technique

In fMRI, there is always a demand to increase sensitivity in MR signal above background noise. Two terms used in the MRI field that relate to signal sensitivity are signal-to-noise ratio (SNR) and contrast-to-noise ratio (CNR). SNR provides a quantitative evaluation of the MRI image before the start of an imaging session. A simple and practical way to measure SNR is to draw two identical region-of-interests (ROI), one over the object and another in the surrounding field of view (Fig. 5). The SNR should be comparable across experiments, a measure of system stability. We routinely perform this calculation in our laboratory at the beginning of each imaging session. CNR is of vital importance because it reflects signal contrast between two functional states. Specifically, CNR is the difference in signal intensity between a stable window of baseline activity and a functional challenge (e.g. drug administration, odour presentation, visual stimulation). Standardised measurements of CNR can be assessed at the beginning of each imaging session by simple block design of (off–on–off–on–off) of 5% CO_2_ inhalation at 1-min intervals.

Anything that will increase SNR and CNR in fMRI will increase spatial and temporal resolution. A simple but costly way of increasing MR signal in functional imaging is to increase the field strength of the main magnet. As an approximation, signal intensity increases linearly as you move up in field strength (15). This hardware solution is not always feasible because ultra-high field scanners are still few in number and not readily available to behavioural neuroscientists as research tools. Consequently, exogenous contrast agents have been developed to achieve high sensitivity even at low field strength scanners. For example, monocrystalline iron oxide nanocolloid (MION) is a contrast agent that, when given intravenously, will dramatically change the microenvironment of spinning hydrogen nuclei causing rapid dephasing (16). As noted earlier, an increase in tissue metabolic activity causes a localised increase in blood flow. Because cerebral blood vessels have some compliance, there is an accompanying increase in blood volume in the area of neuronal activity. The increase in CBV raises the relative concentration of MION, reducing the MRI signal. The magnitude of the change in signal contrast is far greater than the endogenous contrast imaged with BOLD or CBF techniques. Indeed, the major advantage of CBV over BOLD and CBF techniques is its use in fMRI at magnetic field strengths under 3.0 T. However, to take full advantage of the CBV technique, gradient echo pulse sequences must be used which distort the image quality and complicate data interpretation in brain areas sensitive to susceptibility artifacts (Fig. 5).

**FIG. 8:**
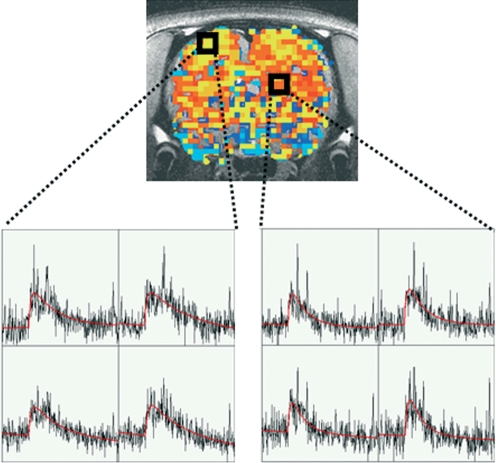
Shown is an activation map for the cerebral blood volume (CBV) response (percent change in area under the curve) in a conscious rat in response to intravenous ethanol (0.75 g/kg). Below are raw time course data from the four contiguous voxels located in each of the black boxes depicted in the activational map. The CBV signal in each voxel was fitted to a nonlinear model using the Analysis of Functional Images (AFNI) software program (29). From these data, it is possible to assess the pharmacokinetic profile of ethanol's action on the brain.

CBV has been used most often in pharmacological MRI to assess brain activation to a sustained drug challenge. With its enhanced CNR, it is possible to assess dose–response and pharmacokinetic relationships with changes in MR signal for discrete brain areas. Presently, work is under way in the laboratory to examine the effect of ethanol on brain activity (17). Using a nonlinear pharmacokinetics model (18), it is possible to fit the raw CBV data to an ethanol-induced response curve, calculate the area under the curve (AUC), and compare this change with a vehicle control (Fig. 8). Three important pharmacokinetic parameters can be extracted using this model: (i) absorption rate; (ii) peak to peak response (maximum response); and (iii) elimination rate. In the data shown, there are no significant differences in the response to ethanol challenge between cortex and striatum in terms of absorption and maximum response; however, a much slower elimination rate was observed in cortex compared to striatum leading to CBV signal with a greater AUC in cortex.

## Methodological considerations when imaging conscious animals

### Controlling for motion artifact

Motion artifact is a considerable problem in fMRI studies. Any minor head movement distorts the image and may also create a change in signal intensity that can be mistaken for stimulus-associated changes in brain activity (19). In addition to head movement, motion outside the field of view caused by respiration, swallowing and muscle contractions in the face and neck are other major sources of motion artifact (20, 21). Motion artifact can be reduced by the use of general anaesthesia. Indeed, historically, the primary reason for imaging fully anaesthetised animals is the reduction in noise leading to improved signal resolution. However, this is not a feasible solution for neuroscientists wanting to understand the neurobiology of behaviour as it precludes the study of brain activity involving cognition and emotion. Furthermore, anaesthetics depress neuronal activity reducing MR signal (6, 22, 23). Consequently, different methods have been developed to restrain animals during an imaging session to minimise motion artifact. Surgery is one option, in which the animal has a head-stabilising post or cranioplastic cap fixed to the skull. During imaging, this skull implant is locked into a holder in the bore of the magnet (24–26). In place of surgery, animals can be paralysed with curare-like drugs and artificially ventilated to minimise motion artifact and to control for the levels of blood gases (27). One entirely noninvasive system developed by Insight Neuroimaging Systems (Worcester, MA, USA) can be used to set up an animal in just a few minutes (28). In brief, just prior to the imaging session, animals are lightly anaesthetised with isoflurane gas. A topical anaesthetic is applied to the skin and soft tissue around the ear canals and over the bridge of the nose. The head is secured into a stereotaxic-like support system with ear bars and nose clamp. The body of the animal is placed into a body restrainer that is mechanically independent from the head holder. This design isolates all of the body movements from the head restrainer and minimises motion artifact. After the animal is set up, the isoflurane gas is removed and the restraining system is positioned in the magnet. Animals are fully conscious within 10–15 min and functional imaging can proceed. Central nervous system activity may, of course, be influenced by residual effects of anaesthesia required for setting up animals in restrainers, however, by using anaesthetics with rapid elimination from the body (such as isoflurane), such issues are minimised. With conscious imaging, at least any effects of the anaesthetic are minor and are temporally removed as far as possible from functional scans, by protocols in which anatomical images are collected first. Thus, in general, in our laboratory, functional scans are carried out at least 30 min after animal subjects have regained consciousness, and are likely to be minimally affected by prior anaesthesia. Determining with certainty whether the use of anaesthesia for setting up restraint affects functional MRI data is difficult because it would be impossible to set up an animal without use of sedation for control comparisons.

**FIG. 9:**
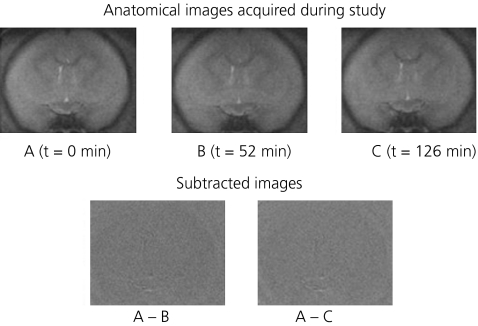
Shown are anatomical images collected over the time from the same brain slice. Voxel by voxel subtraction of these images show no evidence that there was any movement of the head during the imaging study (35).

Following an imaging session there are several simple ways to assess motion artifact: (i) subtraction of anatomical data across the imaging session; (ii) qualitative analysis of time series movies looking for voxel displacement; and (iii) analysis of raw data time series for course spikes. Movement of the head by a single voxel or more over the course of an imaging session will appear as a ‘ghost’ image following subtraction of anatomical data sets (Fig. 9). Time series movies show ‘blips’ or distortions that correlate with course spike activity in the raw data. More elaborate software programs have been written to evaluate and correct for motion artifacts (29).

### Controlling for stress

The stress caused by immobilisation and noise from the MR scanner during functional imaging in fully conscious animals is a major concern. To address this problem, animals are routinely acclimated to the imaging procedure prior to their first scanning session. The acclimation procedure is essentially a simulated scanning session. Animals are anaesthetised with isoflurane as described above for securing the animal into the restrainer. When fully conscious, the animal is exposed to simulated experiment by placing the restraining unit into a black opaque tube ‘mock scanner’ with a tape-recording of an MRI pulse sequence. This procedure is repeated every day for 4 days. Following acclimation, rats show a significant decline in body temperature, motor movements, heart rate and plasma corticosterone levels compared to their first day of restraint (30). Another advantage of acclimating animals for imaging is an increase in CNR. The reduction in motor movement decreases the baseline level of noise resulting in better signal resolution (30). Interestingly, basal blood flow to the brain is not affected by acclimation. There is no difference in basal CBF in animals exposed to their first restraint and imaging session compared to the same animals after several days of acclimation. This finding is particularly important because as noted above, changes in baseline perfusion affect the magnitude of the BOLD response.

The common marmoset monkey (*Callithrix jacchus*) is an excellent subject for fMRI studies. Marmosets acclimate in only 2–3 days and can be imaged while fully conscious without the need for surgery or paralysis to minimise motion artifact (31). Their small size (300–500 g) allows them to be imaged in ultra high field, small bore scanners. Rhesus macaques also prove to be adequate subjects for fMRI because they can be acclimated and trained to relax quietly in a sphinx like position in a horizontal bore (25, 26) or sit up-right in a vertical bore (32). Indeed, Gash *et al*. (33), at the College of Medicine, University of Kentucky, resolved many of the issues associated with imaging fully conscious rhesus monkeys and published a comprehensive description of their equipment and procedures. Frederick *et al*. (34), at McLean Hospital, Brain Imaging Center, recently acclimated and imaged cynomolgus monkeys for up to 2 h using a totally noninvasive restraint device developed by Insight Neuroimaging Systems (Worcester, MA). Monkeys were exposed to both photic and noxious thermal stimulations. Areas of activation corresponded to those activated by the same stimuli in unanaesthetised human volunteers. Motion during these procedures did not exceed < 0.3 mm (translational) and < 0.15° (rotational) during scans. This level of motion is below that typically observed in human studies and was adequately dealt with by motion correction.

### Eliciting behavioural responses in the magnet with natural stimuli

What behaviours can be studied in the magnet and how can they be triggered during an imaging session? Obviously, any behaviour that requires a consummatory act would be very difficult to study with fMRI because the immobilisation alone most likely would prevent the motor response that defines the behaviour. However, fear, anger and hunger are examples of internal states of arousal and motivation. These types of behavioural conditions are fertile areas of investigation using fMRI. An investigator can collect a library of smells and visual images that have proven ethological significance in the animal's natural habitat and in the seminatural environment of the laboratory setting. These smells and images can be used to communicate with the animal in the magnet. For example, we have used odours of novel receptive female marmosets to elicit sexual arousal in male marmosets during an imaging session (35, 36). Vocalisations, while a key form of communication in many animals, are hard to use in imaging studies because of the noise created by the scanner. To date, no method has been developed for using vocalisations to elicit changes in brain activity in conscious animals during an imaging session.

**FIG. 10:**
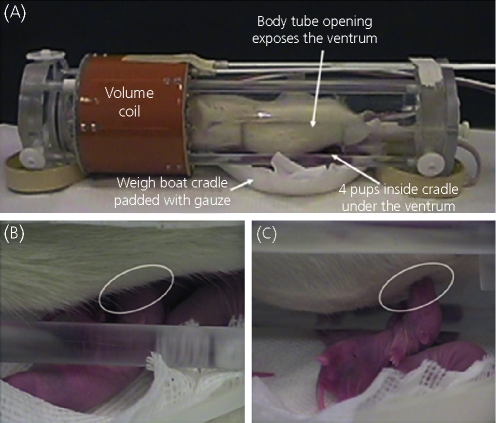
Photographs showing a conscious dam restrained in the device used for functional magnetic resonance imaging. A cradle of the dam's pups is positioned to allow access to the teats for suckling. © 2005 The Society for Neuroscience.

**FIG. 11:**
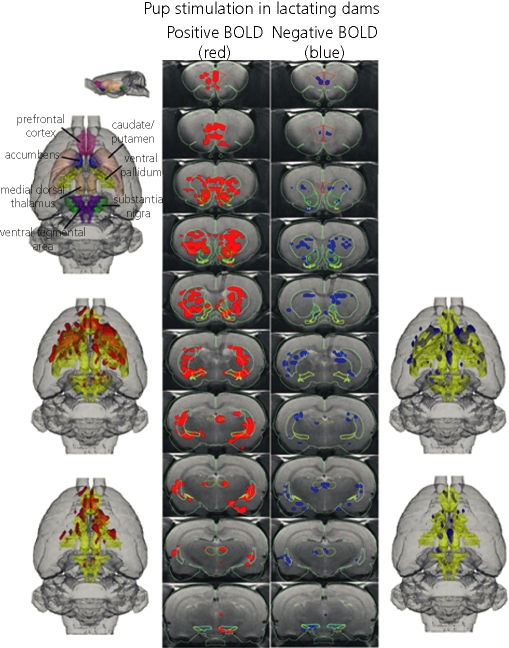
Three-dimensional activational map showing that pup suckling activates the reward system in lactating dams. Upper left picture is a translucent shell of the brain viewed from a dorsal perspective showing colour coded volumes of interest (VOIs) corresponding to anatomical geometries of the mesocorticolimbic and nigro-striatal dopamine systems. Colours have been melded into a single functional VOI (yellow) showing localisation of positive (red) and negative (blue) BOLD signal changes with pup stimulation. The top left brain (below the colour coded shell) includes both dopamine systems while the bottom left brain has masked the caudate/putamen and substantia nigra comprising the nigro-striatal dopamine system revealing the mesocorticolimbic or reward system. Pictures on the right hand side are the corresponding negative BOLD images for each functional volume, respectively. The middle columns show traditional activation maps of contiguous brain sections with labelled regions of interest (37).

Another way of communicating with animals in the magnet is to bring the natural activating stimulus directly into the bore of the magnet. In a recent study, we imaged changes in BOLD signal in dams in response to pup suckling (37). This was accomplished by attaching a cradle with pups to the restrainer beneath the ventrum of the mother. A shield separates the pups from the mother's teats. When the shield is pulled away, the pups immediately begin to suckle (Fig. 10) resulting in activation of the mesolimbic dopaminergic pathway (Fig. 11). In another example, we built a perforated Plexiglas vivarium that can be fitted into the magnet bore directly in front of the subject being imaged. The vivarium is scented with the bedding of the subject's home cage. Robust changes in brain activity can be elicited by placing different ‘intruders’ into the vivarium (e.g. male competitors or sexually receptive females).

### Identifying discrete behavioural responses

How do we tease apart the elements of a complex behaviour and correlate these changes with brain activity using fMRI? With careful attention to the temporal pattern of behavioural activation, it may be possible to identify and correlate nuances in a complex behaviour with changes in BOLD signal. For example, the presentation of olfactory and visual stimuli of a predator to elicit a fear response in an animal would be expected to activate olfactory and visual pathways, limbic and cortical structures mixed with motor pathways involved in initiating freezing or escape behaviour. Outside the magnet, this stimulus-evoked behaviour could be videotaped, time coded and analysed to yield a record of the sequence and chronology of the behavioural response in the form of a time-event table. With lag sequence analysis, the contingency and timing between subtle behavioural events can be predicted. The fMRI acquisition parameters can be set to provide the temporal resolution necessary to match this time-event table. When combined with well-designed control studies, it may be possible to correlate the spatial and temporal pattern of brain activity with discrete events in the complex behaviour. Indeed, it is routine in our laboratory to run our studies on the bench top before an actual imaging session. For example, in a just completed study, we imaged the neural circuitry contributing to the genesis of generalised tonic/clonic seizure by collecting continuous, high resolution, multislice images at subsecond intervals following administration of an epileptogenic agent (38). Coordinating the timing of the image acquisitions with the predicted onset of seizure was based on electroencephalogram recordings and muscle contractions, performed on the bench top.

### Data analysis

**FIG. 12:**
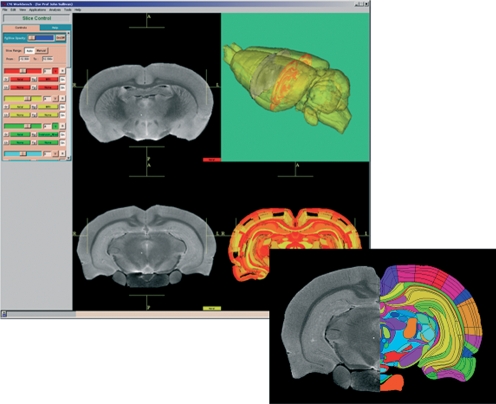
Shown in the background is a graphical interface with both image and geometry data displayed. The left displays are magnetic resonance (MR) imaging image data from a rat brain. The lower right display is geometry-based two-dimensional display of a segmented rat brain corresponding to the registered rat brain image in the lower left corner. The upper right display is a three-dimensional view of the geometry-based rat brain shell. Superimposed within the shell are the MR image on the left and the segmented slice from the bottom of the display. Shown in the lower right is an MR image of a rat brain slice registered to a cross section of the fully segmented atlas.

Although there are many programs available for fMRI data analysis, many of these are aimed at analysing human imaging data. Some programs, including Stimulate (39) and AFNI (29), are readily available and suitable for analysing animal imaging data. Additionally, we have developed our own registration, segmentation, and cumulative analyses tools, now commercially available as MIVA (40). MIVA employs a quantitative analysis strategy, successfully developed and optimised for the rat brain. Each subject is registered or aligned to a fully segmented rat brain atlas that has the potential to delineate and analyse more than 1200 distinct anatomical volumes within the brain. These detailed regions are collected into 96 subvolumes (e.g. dentate gyrus, insular cortex, anterior dorsal thalamus, accumbens) that are grouped into 12 major regions of the brain (e.g. amygdaloid complex, cerebrum, cerebellum, hypothalamus). In addition, the atlas is designed to represent functional neuroanatomical systems, such as the primary and vomeronasal olfactory systems (Fig. 12).

The matrices that transformed the subject's anatomy shells to the atlas space are used to embed each slice within the atlas. All transformed pixel locations of the anatomy images are tagged with the segmented atlas major and minor regions creating a fully segmented representation of each subject. The inverse transformation matrix [**T**_i_]^−1^ for each subject (i) is also calculated. An interactive graphic user interface facilitates these shell alignments (41). Approximately 15–20 min per subject are required to create the slice perimeters, run the marching cube, align the geometries and create the final segmented anatomy.

Statistical t-tests are performed on each subject within their original coordinate system. The control window is a defined set of image acquisitions over the first 3–5 min. The stimulation window is a defined set of image acquisitions lasting from 5 to 30 min. The imaging is continuous without interruption. The t-test statistics use a 95% confidence level, two-tailed distributions and heteroscedastic variance assumptions. Due to the multiple t-test analyses performed, a false-positive detection controlling mechanism is introduced (42). This subsequent filter guarantees that, on average, the false-positive detection rate is below our cutoff of 0.05. These analysis settings provide conservative estimates for significance. Those pixels deemed statistically significant, retain their percent change values (stimulation mean minus control mean) relative to control mean. All other pixel values are set to zero.

## Applications in behavioural neuroscience research

### Brain/environment interactions during development

There are myriad examples in animal studies showing early emotional or environmental insult can affect brain development with long-term neurobiological and behavioural consequences. Insights into the aetiology of mental illness and drug addiction may be gleaned by longitudinal studies examining the interaction between a vulnerable gene pool, a stressful environment, and/or exposure to drugs of abuse. Because fMRI is noninvasive, it can be used to study the same animal over the course of its life. Presently, studies are underway in the laboratory examining the psychosocial and cognitive effects of methylenedioxymethamphetamine (MDMA) given orally to adolescent marmoset monkeys in doses that mimic recreational drug use in human adolescents. Functional imaging is used to follow the changes in brain activity in response to acute and chronic MDMA exposure as monkeys grow from adolescence into adulthood (43). Functional imaging can also be used to study animal models of aspects of drug addiction, such as progressive changes in response to repeated doses [whether increased responding (i.e. sensitisation) or decreased responding (i.e. tolerance)]. Repeated administration of drugs of abuse modifies behavioural responses in rats under many experimental conditions. Alterations in drug-induced behaviour over time are usually measured as increases in locomotor activity or stereotypic behaviour, preference for a drug-conditioned environment, or reinforcement of drug self-administration. For example, daily administration of a single cocaine dose progressively enhances locomotor behaviour in rats termed behavioural sensitisation. Although the initial actions of cocaine occur primarily within the mesolimbic dopamine system, there is a growing body of studies showing that other brain regions and many other neurotransmitter systems are involved in the long-term response to this drug. Imaging the progressive changes in brain activity following repeated administration of cocaine or other drugs of abuse permits an integrated view of the same animal over time during its transition to a sensitised state.

We recently assessed the effects of acute and chronic cocaine administration on brain activity in awake, acclimated male rats (44–46). Our data showed that an intracerebroventricular dose of 20 µg of cocaine produces increases in BOLD signal in areas corresponding to the brain mesocorticolimbic or reward pathway. This dose of cocaine also elicits increases in locomotor activity and dopamine metabolism in animals studied outside the magnet. Rats administered cocaine repeatedly for one week before imaging showed only modest increases in brain BOLD activity when given a challenge dose during functional imaging. It appears that with chronic administration brain activity in response to cocaine is reduced as compared to the first exposure, an effect akin to pharmacological tolerance. To assess whether these changes in neural responsiveness to cocaine with chronic exposure corresponded to altered cerebrovascular reactivity, animals were given a very brief hypercapnic challenge in order to stimulate a ‘mock’ BOLD response in the absence of neuronal activity. We found no differences in haemodynamic-related BOLD responses between acute and chronically treated animals, suggesting altered brain responsiveness to cocaine instead. Results from these studies showed remarkable spatial concordance with previous studies looking at cocaine stimulation of metabolic activity using the 2-deoxyglucose autoradiography technique (47).

### Imaging the neural circuitry of emotion

Three areas of behaviour that carry high emotional valence are fear, sexual arousal, and aggression. Much has been learned about the neural circuitry involved in these emotions from imaging human volunteers (48). However, there are limitations in the types of experiments that can be performed on humans. Moreover, many human studies are confounded by the heterogeneity of the population sample and the psychosocial history of the volunteers. Consequently, this is an exciting area of study using animals. However, the biggest problem in imaging emotions in animals is data interpretation. Although humans can provided a semiquantitative measure of their emotions using visual analogue scales and other assessment methods, this level of communication is absent in animal imaging.

To evaluate brain activity associated with sexual arousal, we imaged male marmoset monkeys during presentation of vaginal odours of receptive females versus odours from ovariectomised females (35, 36). Again, prior to the imaging session, we studied the stimulus response pattern of male marmosets during exposure to periovulatory odours of novel females. Measures of sexual arousal were collected and correlated with changes in physiology and endocrinology (49). The anticipated changes in autonomic physiology were corroborated during presentation of periovulatory odours during an imaging session. Interestingly, a common neural circuit comprising the temporal and cingulate cortices, putamen, hippocampus, medial preoptic area and cerebellum shared showed positive BOLD response to peri-ovulatory odours but negative BOLD response to odours of ovariectomised females. The negative BOLD data were interpreted as a reduction in brain activity (for more on interpreting negative BOLD data, see response to question 4 in ‘Questions for the bench’, below). These data suggest the odour driven enhancement and suppression of sexual arousal affect neuronal activity in many of the same general brain areas. These areas included not only those associated with sexual activity, but also areas involved in emotional processing and reward.

Nursing has reciprocal benefits for both mother and infant helping to promote maternal behaviour and bonding. To test the ‘rewarding’ nature of nursing, fMRI was used to map brain activity in lactating dams exposed to their suckling pups versus cocaine (37). Suckling stimulation in lactating dams and cocaine exposure in virgin females activated the dopamine reward system. By contrast, lactating dams exposed to cocaine instead of pups showed a suppression of brain activity in the reward system. These data support the notion that pup stimulation is more reinforcing than cocaine, underscoring the importance of pup-seeking over other rewarding stimuli during lactation.

Following these studies, we hypothesised that oxytocin, released in the maternal brain during breastfeeding, may be involved mechanistically. To follow-up the above studies, we exposed postpartum dams to pup suckling before and after administration of an oxytocin receptor antagonist; we also administered oxytocin alone to a control group of dams. Dams exposed to oxytocin showed brain activation that was extremely similar to that activation seen when dams were suckling pups. The brain activation seen in suckling dams was almost completely attenuated by the oxytocin antagonist. Overall, data suggest that oxytocin strengthens mother-infant bonding through acting on key brain areas for reward, emotion, and olfactory discrimination; namely, the nucleus accumbens, prefrontal cortex, ventral tegmental area, amygdala, insular cortex, several cortical and hypothalamic nuclei and the olfactory system (50).

### Investigating effects of psychotherapeutics

Functional MRI shows incredible potential for probing the effects of pharmacological agents on brain activity. Not only does fMRI allow us to understand the fundamental mechanisms of brain function, but it also allows the testing and *in vivo* investigation of the neural actions of potential brain medications. Both acute and long-term progressive effects of drug treatment on brain function can be evaluated using fMRI. The study by Hagino *et al*. (24), examining changes in brain activity following acute and prolonged exposure to dopamine receptor agonists and antagonists, is an obvious application of fMRI in animal studies. For example, serotonin reuptake inhibitors cause a prompt increase in brain levels of serotonin and changes in behaviour (51). Nonetheless, patients treated for depression or obsessive compulsive disorder with serotonin reuptake blockers require weeks of treatment before reporting an improvement in their condition. This would suggest drug efficacy for the treatment of mental illness is due to secondary changes in the serotonin system or other interrelated neurochemical signals and pathways that are slowly affected by the continuous exposure to elevated serotonin with chronic drug treatment. With fMRI, the same animal can be imaged over long periods of time following progressive changes in brain activity associated with continuous drug treatment.

Currently, our group is using the ability of fMRI to follow progressive changes within the same animal to investigate the therapeutic mechanisms of antipsychotic drugs. Similar to selective serotonin reuptake inhibitors (SSRIs), antipsychotic medications require several weeks of administration to exert a therapeutic effect; unlike SSRIs, multiple drugs with radically different pharmacological profiles have demonstrated an ability to abate the positive symptoms of psychotic disorders such as schizophrenia with sustained administration. By using fMRI of pharmacological challenges to specific neurotransmitter systems, we are investigating the changes in neural signalling that occur within the same animal following several weeks of treatment with neuroleptic medications. Correlated with changes in behaviour, the localisation of changes in neural activation resulting from a dopaminergic challenge that appear after sustained treatment with neuroleptic medications may provide a marker of therapeutic action, and may facilitate the future development of novel medications with reduced side-effects or enhanced efficacy.

With respect to the above cited studies, knowledge on the systemic effects of drugs is paramount for fMRI. Intravenous or intraperitoneal administration of psychostimulant drugs such as cocaine and amphetamine can produce physiological disturbances that hamper fMRI data. Also, some drugs have direct effects on the vascular endothelium in the brain, possibly affecting haemodynamic responses to brain activity which provide the basis for the BOLD signal. These physiological perturbations can be overcome by prior benchtop work to determine dose-effects on systemic physiology of animals. Testing different doses and routes of administration that can eliminate or delay the onset of systemic effects (i.e. cerebroventricular, oral, subcutaneous) can be used in animals and can help improve the resulting fMRI data.

Such studies of drugs of abuse and psychotherapeutics are of relevance for neuroendocrinologists as they illustrate the methodological possibility of administering drugs interacting with neuroendocrine systems (via numerous routes of administration) and following brain activational responses.

### Testing cognitive performance

Deficits in learning and memory are recognised as components of the symptomology of several mental disorders such as attention-deficit disorder (52) and schizophrenia (53). Many learning paradigms do not require any signs of overt behaviour, making them amenable to testing with fMRI. Because animals will readily respond to peripheral stimulation when in the magnet for fMRI, they may be used in studies of classical conditioning. For example, foot shock can be used as an unconditioned response in associative learning paradigms. When coupled with a conditioned stimulus such as light, it can be used in learning studies examining discrimination and perception. Operant conditioning would be more difficult because a behavioural action (e.g. bar pressing eliciting rewarding or punishing stimuli) would be necessary. However, a study by Logothetis *et al*. (32) demonstrated that awake rhesus monkeys can be trained to press buttons during MRI protocols. These advances in the use of conscious animals open the area of cognitive neuroscience to investigation with fMRI. Because many hormones are involved in cognitive change resulting from ageing and/or disease processes, the development of the ability to image cognitive function in model animals whose neuroendocrine status can be manipulated is an important development.

### Limitations and advantages of functional MRI

Functional MRI is a new method available to behavioural neuroscientists to help study the brain. Although there are different fMRI methods, they all involve a change in blood flow to achieve a change in signal contrast. The change in blood flow is coupled to an increase or decrease in brain metabolism. Consequently, from the onset of a stimulus, there is a temporal delay of 2–3 s (54) for BOLD and CBF contrast and even longer for CBV contrast. To achieve a statistically reliable change in signal following a stimulus, it is necessary to average multiple data acquisitions collected over 1–2 min. Imaging contrast that depends on haemodynamic changes will never achieve the temporal resolution of electrophysiology. Therefore, it is not possible to image the initial activation of a behavioural neural circuit in real time. Instead, you are left with a ‘haemodynamic finger print’ of the stimulus response a few minutes after its onset.

Spatial resolution in fMRI is a function of field strength and the radiofrequency electronics. A majority of the data thus far reported in animal studies ranging from mice to monkeys, employed a 64 × 64 matrix with an in-plane resolution of 400–500 µm^2^. When overlaid into a segmented atlas with a resolution of 50–100 µm^2^, it is possible to see patterns of activation associated with many nuclear areas. However, a clear delineation of discrete functional neuroanatomical subgroups, such as those described in the amygdaloid complex (> 20 discrete areas), has not been realised in either animals or humans. Indeed, fMRI is not capable of identifying functional changes in single neurones *in vivo*. However, this spatial limitation is not perceived as a problem because many neurones in discrete locations and of a similar phenotype behave as an ensemble with a coordinated pattern of activation. With greater magnetic field strengths and improved electronics becoming available in the future, it may be possible to parcel out the functional activity of the many discrete nuclei that subdivide the major areas of the brain.

Functional MRI has two features that distinguish it from other methods in behavioural neuroscience research. First, it can be entirely noninvasive. No surgery is required in preparation for imaging, and it is not necessary to euthanise the animal after imaging. Animals can be acclimated to imaging procedures, minimising the stress. A single animal can be imaged multiple times over the course of its natural life. Because neuroadaptation is a developmental process critical to understanding behaviour, fMRI functions as a window to the brain, enabling scientists to follow changes in brain activity in response to factors such as age, environment, hormones, and drugs of abuse. Second, fMRI has the spatial and temporal resolution to resolve patterns of neuronal activity across the entire brain in less than a minute. Synchronised changes in neuronal activity across multiple brain areas can be viewed as functional neuroanatomical circuits coordinating the thoughts, memories and emotions for particular behaviours.

## Questions for the bench

### Question 1: For an investigator interested in behavioural neuroendocrinology, what are key steps needed to establish fMRI techniques as part of ones armamentarium of tools?

Start by forming collaborations with existing imaging groups, either in your immediate area or further afield. Some groups are setting up virtual imaging centres, so that you can, in effect, run their magnet long-distance. If hoping to develop your own imaging resources, developing intrainstitutional collaborations in order to accumulate a critical mass of interested researchers is very important. The availability of ‘lots of space’ is becoming less and less necessary, as imaging technology becomes smaller, with active shielding. Costs are also lower than they were, and continue to drop. Inter-institutional collaboration will also add strength, especially if other institutions bring on board engineering or physics expertise to add to your applications expertise. Information technology support will be important. Consider service and maintenance contracts with equipment providers unless there is considerable MR expertise at your institution.

### Question 2: What level of expertise is needed to attain to understand and make intelligent use of the data? Are there particular academic courses that would be useful to provide background or advanced knowledge?

With respect to running equipment for studies, the manufacturing companies give tutorials on use of their hard- and soft-ware. To interpret the data that you obtain, a good understanding of where the data have originated (i.e. an understanding of basic MR principles such as where the BOLD signal comes from) will be essential. Good introductory materials (55) will get everyone in the laboratory up to speed on the basics. Several institutions offer basic introductory MR physics courses that can be very useful, so check out local colleges for information. Conferences such as ISMRM (International Society for Magnetic Resonance in Medicine) often hold sessions providing introductions to the field, and more advanced sessions bringing scientists up to date on specific topic areas. Bear in mind that data interpretation is fluid and developing in the field, so you need to keep on top of current literature and perspectives.

### Question 3: What are the key elements in hardware and software to look for in choosing to enter the field?

Assuming most readers will be neuroendocrinologists (i.e. applications driven and not MR physicists), it is important to go for a user-friendly, plug and play machine. Our program consensus is that a 7T actively shielded animal magnet offers the ideal combination of usability and affordability at the moment, providing enough field strength to enable good MR spectroscopy as well as functional and anatomical imaging, whilst not costing as much as higher field strength systems. Software decisions need research. There are useful public domain software programs, in addition to software available for purchase. Bear in mind that many MR software analysis programs are pitched at human imaging, and include features that are unnecessary or irrelevant for animal imaging.

### Question 4: Most of the BOLD signals discussed are positive; however, one was negative. What are issues involved in interpreting negative BOLD signals?

Interpretation of negative BOLD data remains controversial. Use of the terms ‘reduced neural activity’ and ‘deactivation’ are generally used to describe negative changes in the brain BOLD activity as compared to baseline activity. The term is not necessarily meant to explain the nature of these changes (changes in blood flow or the firing of neurones). Several lines of evidence show that the negative BOLD reflects one of three possible scenarios: (i) the rate of oxygen consumption (CMRO_2_) in areas of high synaptic and neuronal activity exceeds compensatory blood flow; (ii) ‘vascular steal’ (i.e. preferential flow to the most metabolically active areas leaving the abandoned areas facing less blood flow in the presence of a constant CMRO_2_); and (iii) a reduction in brain activity and blood flow from basal levels. Discussion continues in the field, although there is evidence to support the idea that negative BOLD does indeed correlate with a decrease in neuronal activity. For example, it has been demonstrated that decreases in local field potentials and multiunit activity occur simultaneously with negative BOLD signal in monkey visual cortex (56). In human studies, negative BOLD has also been shown to be linked primarily to decreased neural activity with possibly a smaller component resulting from local cerebrovascular changes that were not neural activation dependent (57).

### Question 5: What major innovations are expected to occur in this field in the next 5–10 years?

A major innovation in MR that would impact the fields of behavioural neuroscience and neuroendocrinology would be the development of paramagnetic ligands for molecular imaging. Paramagnetic ligands are under development that can localise and cluster at cell surface receptors, or following receptor interaction, become internalised and accumulate in cells with specific phenotypes representing different neurotransmitter systems. Essentially, you could localise and quantify receptor density for different chemical signals in the brain. This innovation would bring the advantage of PET imaging to MRI but with the added benefit of greater spatial and temporal resolution.
